# CDK4/6 inhibitor palbociclib suppresses IgE-mediated mast cell activation

**DOI:** 10.1186/s12967-019-2026-9

**Published:** 2019-08-20

**Authors:** Yi-Bo Hou, Kunmei Ji, Yue-Tong Sun, Li-Na Zhang, Jia-Jie Chen

**Affiliations:** Department of Biochemistry and Molecular Biology, School of Medicine, Shenzhen University, Shenzhen, 518060 People’s Republic of China

**Keywords:** Mast cells, Palbociclib, CDK inhibitor, Drug repurposing

## Abstract

**Background:**

Mast cell activation causes degranulation and release of cytokines, thereby promoting inflammation. The aim of this study was to investigate the inhibitory effect of CDK4/6 inhibition on mast cell activation in vitro and in vivo.

**Methods:**

RBL-2H3 rat basophilic leukemia cells (BLCs) and mouse bone marrow-derived mast cells (BMMCs) were sensitized with anti-dinitrophenol (DNP) immunoglobulin (Ig)E antibodies, stimulated with DNP-human serum albumin (HSA) antigens, and treated with the CDK4/6 inhibitor palbociclib. Histological stains were applied to reveal cytomorphological changes. Murine IgE-mediated passive cutaneous anaphylaxis (PCA) and ovalbumin (OVA)-induced active systemic anaphylaxis (ASA) models were used to examine palbociclib effects on allergic reactions in vivo. Western blots were performed to detect the expression of cell signaling molecules associated with mast cell activation.

**Results:**

Activated BLCs and BMMCs released copious granule-related mediators (histamine and β-hexosaminidase), which was reduced by palbociclib in a concentration-dependent manner. Palbociclib inhibited expression of the mast cell activation marker CD63 in activated BLCs and inhibited granule release (visualized with toluidine blue staining) while preventing morphological changes, (elongated shape maintained) and filamentous actin (F-actin) reorganization. Palbociclib suppressed molecular Lyn and/or mitogen-activated protein kinase (MAPK) signaling associated with mast cell activation in stimulated BLCs and attenuated allergic reactions in PCA mice dose dependently. Palbociclib attenuated body temperature reduction and diminished serum histamine levels in ovalbumin OVA-challenged ASA mice.

**Conclusion:**

Palbociclib suppresses IgE-mediated mast cell activation in vitro and in vivo, suggesting that it may be developed into a therapy for mast cell-mediated allergic diseases via inhibition of mast cell degranulation.

## Introduction

Common allergic diseases, including asthma, allergic rhinitis, and specific dermatitis, are consequent to hypersensitive immune reactions [1]. In a given year, approximately one in five people in the world are affected by allergic diseases [2]. Socioeconomic development has been associated with an increasing incidence of allergic diseases year over year [3, 4]. Importantly, mast cells, which are major innate immunity effector cells, play a principal role in inducing allergic inflammation by releasing various mediators, including lipid mediators, chemokines, and cytokines [5]. Thus, mast cells are an attractive target for the treatment of allergic inflammation.

Mast cell activation, which plays a key role in inducing IgE-mediated allergic inflammation, depends on cross-linking of antigen immunoglobulin (Ig)E complexes with the high affinity IgE receptor, commonly referred to as FcεRI, on the surface of mast cells [1, 6]. The subsequent mast cell degranulation that ensues can trigger acute inflammatory reactions and promote chronic allergy progression by secreting histamine, proteases, and chemotactic factors, as well as by engaging in de novo synthesis of inflammatory cytokines [5, 7]. During an acute allergic response, histamine, which is a well-established vasodilator, also acts to increase vascular permeability, leading to a low body temperature and leukocyte extraversion from the circulation into local tissues [8]. Therefore, suppression of mast cell activation has the potential to attenuate allergic inflammation [9].

Antihistamine and steroid drugs are common clinical therapies used to treat allergic diseases [10, 11]. Additionally, small molecule inhibitors targeting leukotrienes or histamine receptors have been developed to treat allergic diseases [12]. Mast cell stabilizers that inhibit activated mast cell release (e.g. sodium cromoglycate, nedocromil, and lodisa) have emerged as another potential allergy treatment approach [13, 14]. Whereas these treatments target allergy symptom control, blockade of mast cell activation represents an opportunity to alleviate the immune dysfunction underlying allergic diseases more directly [15].

Palbociclib (IBRANCE; PD0332991; Pfizer; C_24_H_29_N_7_O_2_) is an orally available drug approved by the US FDA for the treatment of cancers [16]. Notably, it was approved as a first-line treatment of estrogen receptor-positive (ER+)/human epidermal growth factor receptor 2-negative (HER-) advanced breast cancer based on PALOMA-1 study findings [16, 17]. Palbociclib, is a selective cyclin-dependent kinase (CDK)4/6 inhibitor, with low enzymatic half-maximal inhibitory concentrations for CDK4 (11 nM) and CDK6 (15 nM), that inhibits retinoblastoma protein phosphorylation in early G1 phase, leading to cell cycle arrest and thus suppression of cell proliferation [17].

The effects of CDK4/6 inhibitors, such as palbociclib, on mast cell activation and allergic reactions remain to be clarified.

The aim of this study was to investigate potential anti-allergic effects of palbociclib on IgE-mediated mast cell activation. We sensitized mast cells with anti-dinitrophenol (DNP) IgE antibodies and then used DNP-human serum albumin (HSA) antigen stimulation to activate the sensitized mast cells in vitro. We used a murine IgE-mediated passive cutaneous anaphylaxis (PCA) model and ovalbumin (OVA)-induced active systemic anaphylaxis (ASA) model to examine the effects of palbociclib on allergic reactions in vivo. Finally, we explored the molecular mechanisms underlying palbociclib effects on IgE-mediated mast cell activation.

## Materials and methods

### Reagents and antibodies

Palbociclib was purchased from Med Chem Express (Monmouth Junction, NJ). Monoclonal DNP-specific IgE, DNP-HSA, and 4-nitrophenyl N-acetyl-β-D-glucosaminide were obtained from Sigma-Aldrich (St. Louis, MO). Evans blue, formamide, toluidine blue and mast cell stabilizer ketotifen were obtained from Dalian Meilun Biotechnology Co. Ltd. (Dalian, China). Antibodies targeting the tyrosine-protein kinase Lyn, Tyr397 phosphosphorylated (p)-Lyn, mitogen-activated protein kinase (MAPK) p38, c-Jun N-terminal kinase (JNK) (Abcam, Cambridge, MA), extracellular signal-regulated kinase (ERK)1/2, p-p38 (Thr180/Tyr182), p-JNK (Thr183/Tyr185), glyceraldehyde 3-phosphate dehydrogenase (GAPDH; Santa Cruz Biotechnology, Santa Cruz, CA), and p-ERK1/2 (Thr202/Tyr204) (p-ERK1/2) (Cell Signaling Technology, Beverly, MA) were used. Fluorescein isothiocyanate (FITC)-phalloidin was purchased from Yeasen Biotech Co. Ltd. (Shanghai, China). Lyn inhibitor Bafetinib, ERK inhibitor U0126, JNK inhibitor SP600125 and p38 inhibitor SB203580 were purchased from MedChem Express (Monmouth Junction, NJ, USA). Silencing RNAs (SiRNAs) for knocking down the expression of Lyn were designed and obtained from Gene Pharma (Shanghai, China).

### Animals

Female BALB/c mice (4–5 weeks old) were purchased from Guangdong Medical Laboratory Animal Center (Foshan, China), and housed in a specific pathogen-free environment with a relatively stable temperature (24 ± 1 °C) and humidity (55 ± 10%) for 1 week before experimentation. The mice were used to isolate bone marrow-derived mast cells (BMMCs) as well as for our PCA and ASA models. All studies involving mice were performed according to protocols approved by the Animal Care and Use Committee of the School of Medicine of Shenzhen University.

### Cell culture

RBL-2H3 rat basophilic leukemia cells (BLCs; Cellcook Biotechnology Co., Guangzhou, China) were cultured in complete Dulbecco’s modified eagle medium with 4.0 mM l-glutamine with sodium pyruvate penicillin (100 U/ml), 100 μg/ml streptomycin, non-essential amino acids, and 10% fetal bovine serum in a humidified incubator at 37 °C, 5% CO_2_. Mouse bone marrow-derive mast cells (BMMCs) were isolated from BALB/c mouse femurs and cultured in complete RPMI-1640 with 100 U/ml penicillin, 100 μg/ml streptomycin, 2 mM l-glutamine, 1 mM sodium pyruvate, 10 mM 4-(2-hydroxyethyl)-1-piperazineethanesulfonic acid, 10% fetal bovine serum, and 10 ng/ml interleukin (IL)-3 and 10 ng/ml stem cell factor. After 4–6 weeks in culture, mast cell purity reached ≥ 95%, as indicated by flow cytometry detection of cell-surface CD117 and FcεRI expression [18, 19].

### Measurement of β-hexosaminidase

BLCs and BMMCs were dispensed into 24 well-plates, sensitized with 50 ng/ml anti-DNP IgE for 12 h, and washed with Tyrode’s buffer. Palbociclib or other small molecule inhibitors (Lyn inhibitor bafetinib, ERK inhibitor U0126, JNK inhibitor SP600125 and p38 inhibitor SB203580) was then applied for 1 h. Subsequently, after stimulating with 100 ng/ml DNP-HSA for 30 min, the supernatant and cell lysate were reacted for 1.5 h with 1 mM -nitrophenyl-*N*-acetyl-β-d-glucosaminide at 37  °C, followed by quenching with 150 μl carbonate buffer. Optical density (OD) at 405 nm was measured with microplate reader (Bio-Rad, USA) [19–21]. We used the following equation to calculate percent β-hexosaminidase release (% degranulation):

$$ \frac{{\left( {\text{Experimiental release} - \text{Tyrode's release}} \right)}}{{\left( {\text{Triton-X-100\,release} - \text{Tyrode's release}} \right)}}{ \times 100}. $$

### Cell viability assay

BLCs (2  ×  10^3^/well) and BMMCs (1 ×  10^4^/well) were cultured in 96-well plates for 24 h and then treated with palbociclib for 24 h (as above). Cell viability was measured with Cell Counting Kit 8 (Med Chem Express, Monmouth Junction, NJ) according to the manufacturer’s protocol.

### Histamine release measurement

BLCs were placed in 24 well-plates (2  ×  10^5^ cells/well), sensitized with 50 ng/ml anti-DNP IgE for 12 h, washed twice with Tyrode’s buffer, and then treated with experimentally indicated concentrations of palbociclib for 1 h. After stimulating with 100 ng/ml DNP-HSA for 20 min, histamine levels in culture media were determined with enzyme-linked immunosorbent assay (ELISA) kits (IBL, Germany), according to the manufacturer’s protocol [21].

### Flow cytometry

CD63 expression can be used as an index of mast cell degranulation [22]. After stimulation with DNP-HSA, BLCs pretreated with or without palbociclib were washed with phosphate buffered saline (PBS), labeled with PE-CD63 antibody (Millenia Biotec, Auburn, CA), and detected by flow cytometric analysis (Cytoflex flow analyzer, Beckman Coulter, CA).

### Toluidine blue staining

Toluidine blue staining was used to reveal mast cell activation as evidenced by deposition of metachromatic granules against a pale blue background [23, 24]. BLCs were incubated with 250 μl of 4% paraformaldehyde/PBS for 30 min at room temperature. The fixed cells were stained with 300 μl of toluidine blue dye (1% w/v in 0.9% saline solution, pH 2.5) for 30 min. The stained cells were observed with an inverted microscope (Carl Zeiss, Goettingen, Germany) [23].

### Microfilament staining

Filamentous actin (F-actin) is involved in mast cell degranulation [24, 25]. Because phalloidin binds F-actin specifically, we used FITC-phalloidin staining to observe F-actin changes. BLCs were fixed in 4% paraformaldehyde in PBS for 30 min, then washed twice with PBS and permeated with 0.1% T-X100 PBS for 3 min. After washing with PBS, cellular F-actin was stained with 100 nM FITC-phalloidin for 30 min. Cells were imaged with a fluorescence microscope (Carl Zeiss, Goettingen, Germany) via a FITC channel (excitation/emittance = 496/516 nm) [25].

### SiRNA transfection

Rat Lyn-specific siRNA sequences with the following sequences were designed and synthesized by GenePharma Co. (Shanghai, China): 5′-GGACAUAACAAGGAAA GAUTTAUCUUUCCUUGUUAUGUCCTT-3′(siRNA-1) and 5′-CCAUGGGAUAA AGAUGCUUTTAAGCAUCUUUAUCCCAUGGTT-3′ (siRNA-2). The negative control siRNA sequence was 5′-UUCUCCGAACGUGUCACGUTT-3. These siRNAs were transfected into BLCs with the aid of lipofectamine^®^ RNAiMAX reagent (Invitrogen, Carlsbad, CA) according to the manufacturer’s protocol. Following confirmation of the siRNA effect by western-blotting assay, activated siRNA-transfected BLCs were subjected to β-hexosaminidase assays following experimentally indicated pretreatments.

### Western blot

We used western blots to investigate the effects of palbociclib on the activation of the FcεRI signaling regulatory proteins Lyn and the MAPK signaling proteins p38, JNK, and ERK1/2, which have bene reported to be involved in mast cell activation [26]. BLCs were sensitized with 50 ng/ml DNP-IgE overnight and washed twice with PBS and placed in fresh media. After incubating with or without palbociclib at 37 °C for 1 h, BLCs were stimulated with 100 ng/ml DNP-HSA and then washed twice with PBS. Cells were lysed in RIPA buffer (Beyotime, Beijing, China) with a protease-inhibitor cocktail (Med Chem Express, Monmouth Junction, NJ). Cell lysates were centrifuged at 12,000 rpm for 15 min. Supernatants were mixed with a loading sample buffer (Thermo Fisher Scientific) and denatured by heating for 10 min at 100 °C. Proteins were separated by sodium dodecyl sulfate–polyacrylamide gel electrophoresis and transferred to polyvinylidene difluoride membranes (Merck Millipore, Billerica MA). After incubating with a primary antibody in tris-buffered saline with 0.1% tween 20 buffer that contained 5% bovine serum albumin or skim milk. The membranes were incubated with horse-radish peroxidase-conjugated secondary antibodies for 1 h at room temperature in the same buffer. Chemiluminescence reagents (Meilun, Dalian, China) were applied according to the manufacturer’s protocol.

### PCA model

PCA mice were used to examine the effects of palbociclib on IgE-mediated allergic reaction in vivo. The PCA model is an acute allergic animal model wherein allergic reactions are induced by antigen stimulation of mast cells in ear skin [19, 20]. Briefly, BALB/c mice (female, 4–5 weeks-old, 5 per group) were injected intradermally with 0.5 μg of DNP-IgE in the left ear. After a 24-h infiltration period, palbociclib (25 mg/kg or 50 mg/kg) or ketotifen (100 mg/kg, positive control) dissolved in physiological saline was injected intraperitoneally [19–21]. One hour later, 200 μl of 5 mg/ml Evans blue solution containing 0.1 mg/ml DNP-HSA was administered into the tail vein. The mice were euthanized by cervical dislocation an hour after Evans blue injection. The dye was extracted from dissected ears in 700 μl of formamide for 12 h at 62 °C and quantitated by a spectrophotometer at 620 nm. Ear thickness was measured with a dial thickness gauge.

### ASA model

The ASA model has been used to examine immediate-type hypersensitivity, which has been shown to be strongly associated with mast cells [21]. BALB/c mice (female, 4–5 weeks-old, 5 per group) were sensitized with OVA (100 μg OVA and 2 mg alum adjuvant in 200 μl PBS), by intraperitoneal injection on day 0 and day 7 as described previously [21]. Subsequently, palbociclib (50 mg/kg) and ketotifen (50 mg/kg) injections were given intraperitoneally on days 9, 11, and 13. The animals were challenged with OVA injections following sensitization. On day 14, 200 μg of OVA was injected intraperitoneally, and rectal temperature was measured every 10 min for 90 min. After 90 min, a blood sample was obtained from the tail of each mouse. Blood IL-4 and IL-10 levels were measured by ELISA (Shanghai Huzhen Biotechnology Co., Ltd. Shanghai, China) according to the manufacturer’s protocol.

### Statistical analysis

The data are presented as the means with standard deviations (SDs) of at least three independent experiments for cell experiments and of 5 mice per group for animal experiments. Statistical analyses were performed in Prism 7.0 (GraphPad Software, Inc.). One-way analyses of variance (ANOVAs) and Dunnett’s post‑hoc tests for multiple comparisons were applied to detect inter-group differences with a significance criterion of *p* < 0.05.

## Results

### Palbociclib inhibited mast cell degranulation without cytotoxicity

**Fig. 1:**
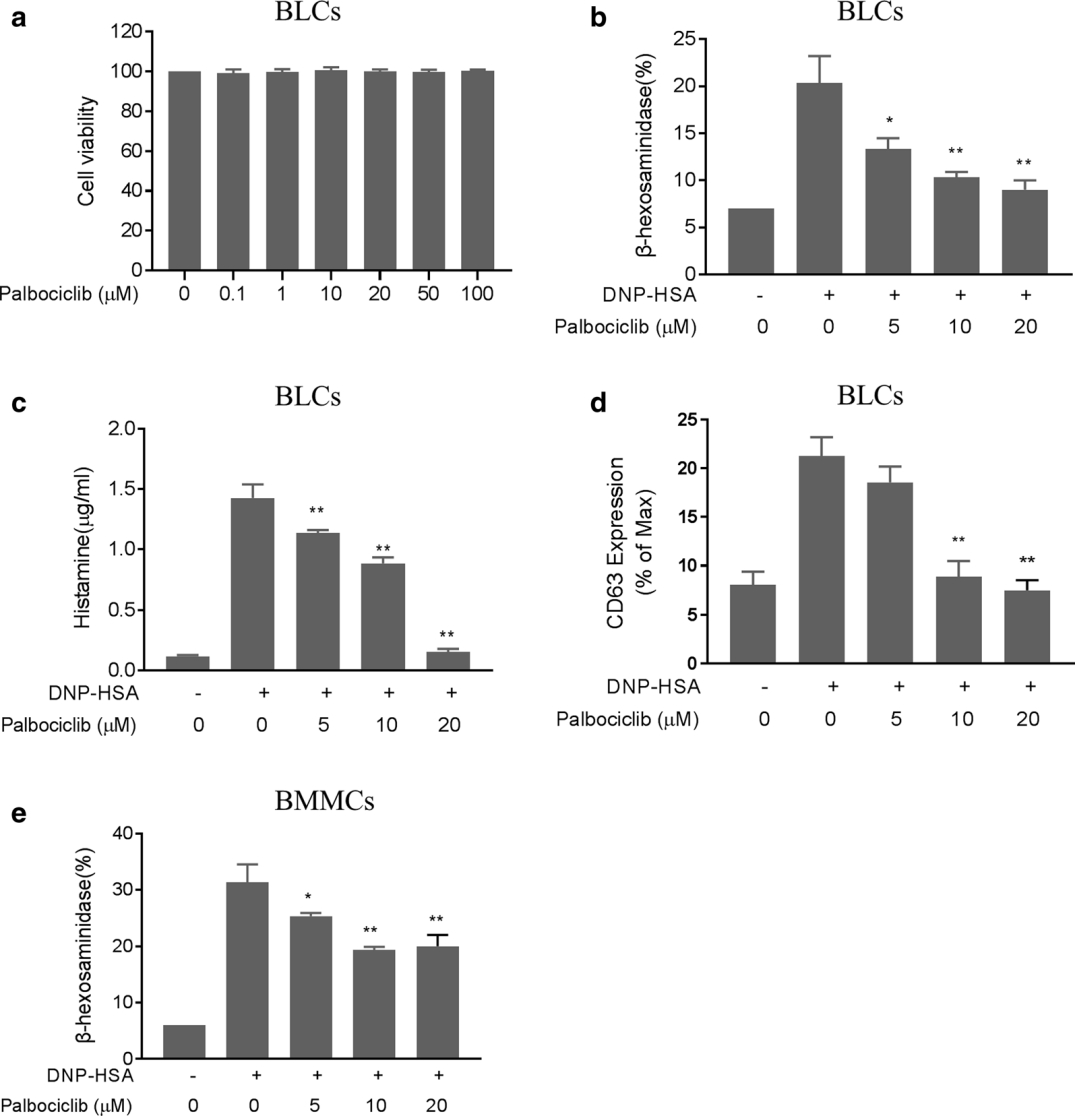
Palbociclib attenuation of mast cell degranulation. **a** Cell viability of B LCs following DNP-HSA challenge with or without 1-h palbociclib pretreatment (BLCs had been anti-DNP IgE-sensitized and incubated with palbociclib for 24 h prior to the challenge). **b** β-Hexosaminidase release from BLCs in **a**. **c** Histamine release from BLCs in A. **d** Flow cytometric analysis of CD63 expression in BLCs. **e** β-Hexosaminidase release from BMMCs stimulated with 100 ng/ml DNP-HSA for 30 min (prior 50 ng/ml DNP-specific IgE priming with or without 1-h palbociclib pretreatment). Means ± SDs of 3 independent experiments are shown; *p < 0.05, **p < 0.01 vs. the HSA-DNP group

Cell viability assays revealed no evidence of palbociclib cytotoxicity in BLCs or BMMCs (Fig. 1a) following 24 h of exposure at concentrations < 100 μM. Palbociclib treatment reduced levels of released granule-related mediators (histamine and β-hexosaminidase) dose dependently in DNP-HSA activated BLCs (Fig. 1b, c). Similar degranulation inhibition was seen in BMMCs treated with palbociclib (Fig. 1e). Cytometric analysis of palbociclib-treated BLCs showed dose-dependent inhibition of the upregulation of the expression of the mast cell activation marker CD63 (Fig. 1d).

### Palbociclib inhibited activation-associated morphological changes in mast cells

**Fig. 2:**
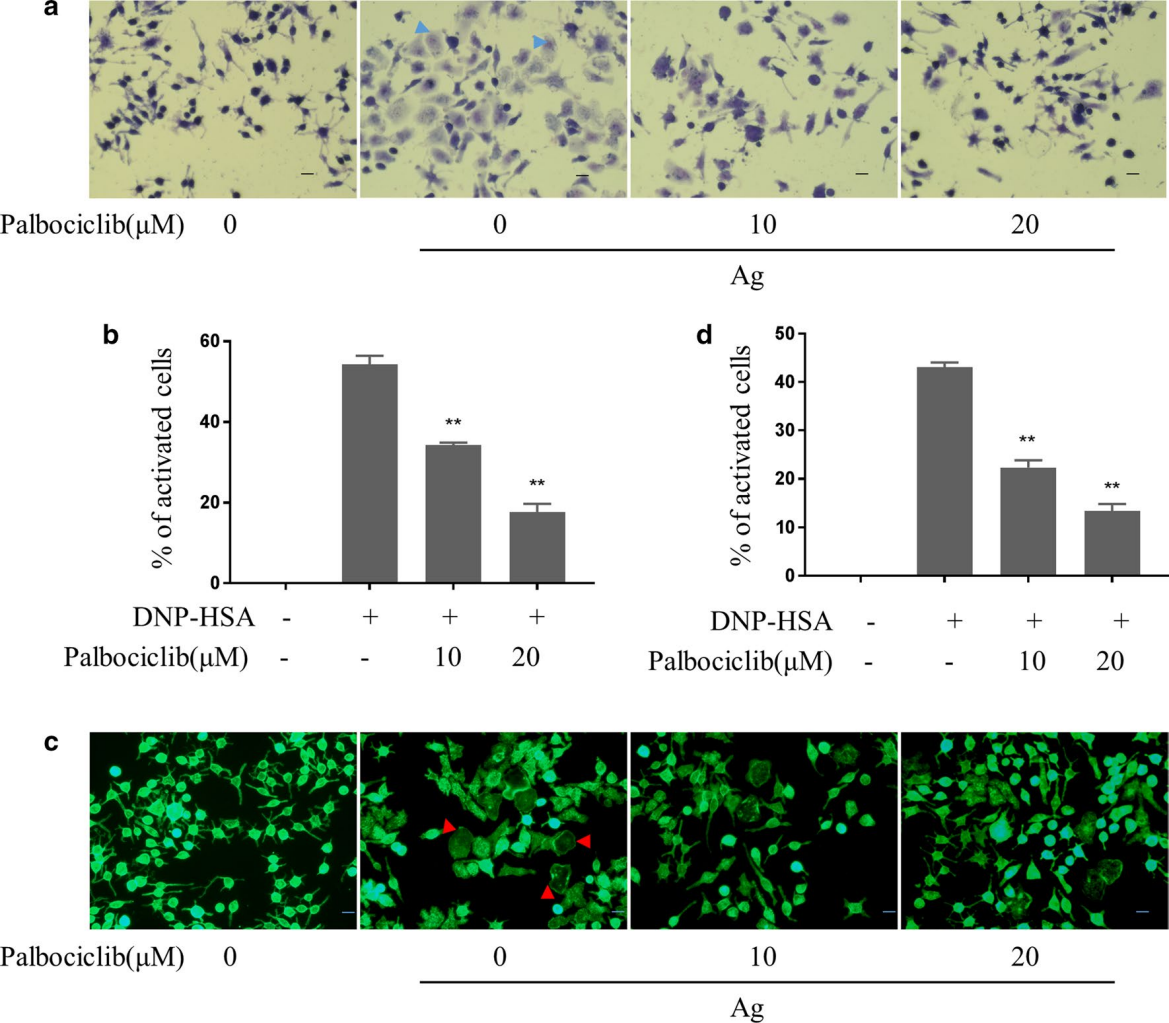
Palbociclib inhibition of morphological changes associated with activation in mast cells. Anti-DNP IgE-sensitized BLCs were pretreated (or not) with palbociclib for 1 h and then challenged with DNP-HSA (100 ng/ml) for 30 min. **a** Toluidine blue-stained RBCs. Blue arrows indicate irregular cell morphology and the release purple particles. **b** The statistical data were from 3 independent experiments. **c** FITC-phalloidin stained BLCs. Red arrow indicates cell morphology irregularity due to decomposition of F-actin cytoskeleton. **d** The data are summarized from 3 independent experiments, *p < 0.05, **p < 0.01, compared to the HSA-DNP group

Non-activated BLCs had an elongated shape with purple intra-cellular particles, whereas activated BLCs had irregular shapes and released purple particles extracellularly. Palbociclib inhibited activation-associated morphological changes and degranulation (particle release) in activated BLCs significantly (Fig. 2a). Non-activated BLCs had a spindle shape and uniformly distributed F-actin. Activated BLCs became elliptical in accordance with their F-actin cytoskeletal changes. Pretreatment with palbociclib inhibited activation-associated shape changes and F-actin cytoskeleton decomposition in activated BLCs (Fig. 2b).

### Palbociclib inhibited signaling pathways involved in mast cell activation

**Fig. 3:**
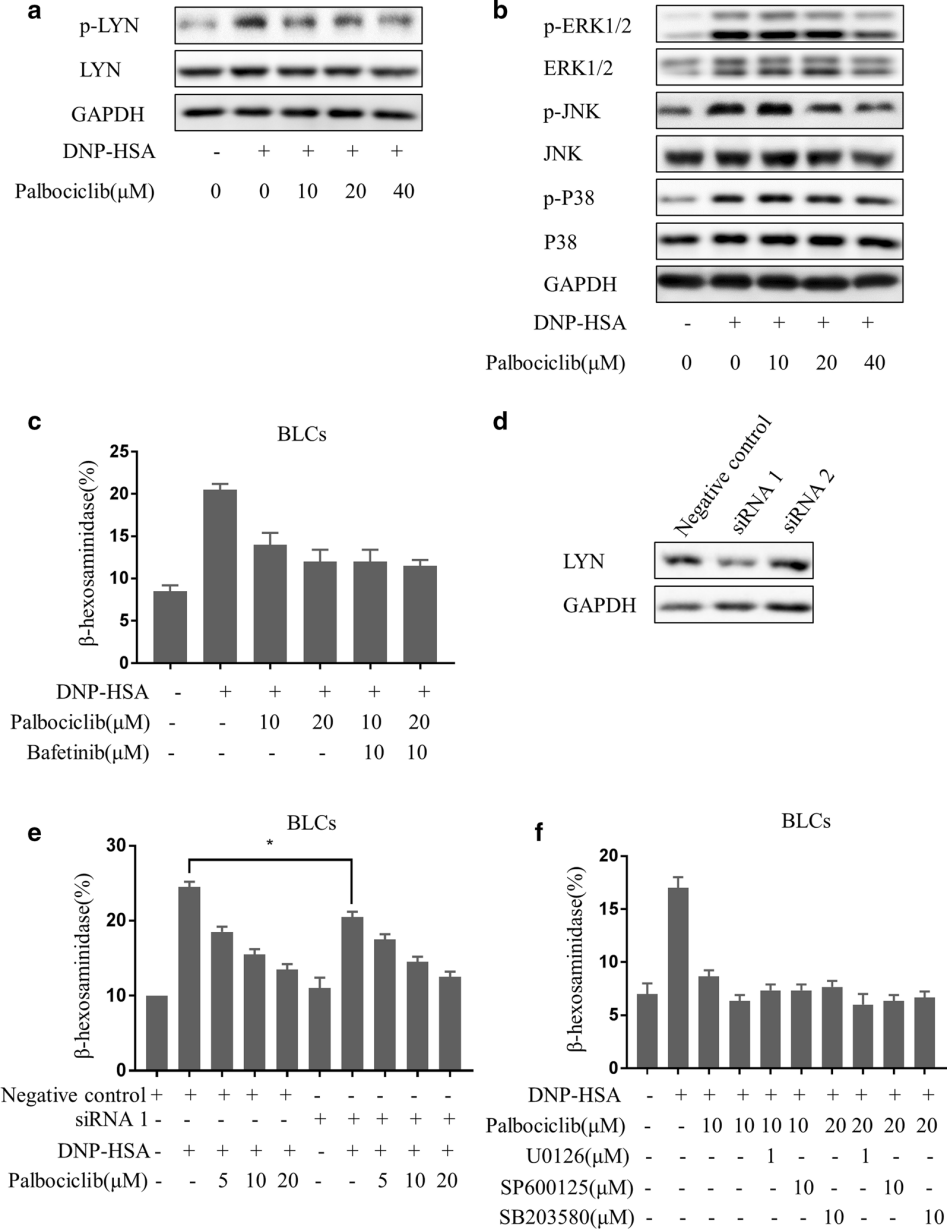
Palbociclib-mediated reduction in levels of phosphorylated proteins that are involved in mast cell activation. Western blots of total lysate samples from activated mast cells (see “Materials and methods”) demonstrating palbociclib treatment (0 μM, 10 μM, 20 μM, and 40 μM) inhibition of Lyn activation (**a**) and MAPK (p38, JNK, and ERK1/2) activation (**b**). **c** β-Hexosaminidase release from activated BLCs subjected to 1-h pretreatments (as experimentally indicated) prior to the challenge. **d** Western blot analysis of Lyn expression in RBL-2H3 cells transfected with siRNA-Lyn. **e** β-Hexosaminidase release from activated BLCs pretreated with siRNA-Lyn and palbociclib. **f** β-Hexosaminidase release from activated BLCs pretreated with signaling inhibitors (ERK inhibitor U0126, JNK inhibitor SP600125, and p38 inhibitor SB203580) for 1 h prior to the challenge

Palbociclib pretreatment, prior to a DNP-HSA challenge, inhibited activation of Lyn as well as activation of MAPKs (p38, JNK, and ERK) in BLCs, as evidenced by decreased levels of p-Lyn, p-p38, p-JNK, and p-ERK1/2 (Fig. 3a, b). The effects of palbociclib on Lyn and MAPK signaling molecules exhibited dose dependence. Experiments with small molecule inhibitors or siRNAs intended to investigate whether palbociclib suppression of mast cell degradation involves down-regulation of Lyn and MAPK signaling showed no statistically significant differences in β-hexosaminidase release levels in DNP-HSA activated BLCs between cells treated with palbociclib alone and cells treated with palbociclib plus the Lyn signaling inhibitor bafetinib (Fig. 3c). Lyn expression was reduced in siRNA-treated BLCs (Fig. 3d). Similarly, down-regulation of Lyn expression in BLCs did not affect palbociclib inhibition of mast cell activation, as evidenced by detection of β-hexosaminidase release (Fig. 3e). Similar results were obtained with the ERK inhibitor U0126, the JNK inhibitor SP600125, and the p38 inhibitor SB203580 (Fig. 3f). These results suggest that palbociclib inhibition of mast cell activation may involve suppression of Lyn and MAPK signaling.

### Palbociclib attenuated PCA in vivo

**Fig. 4:**
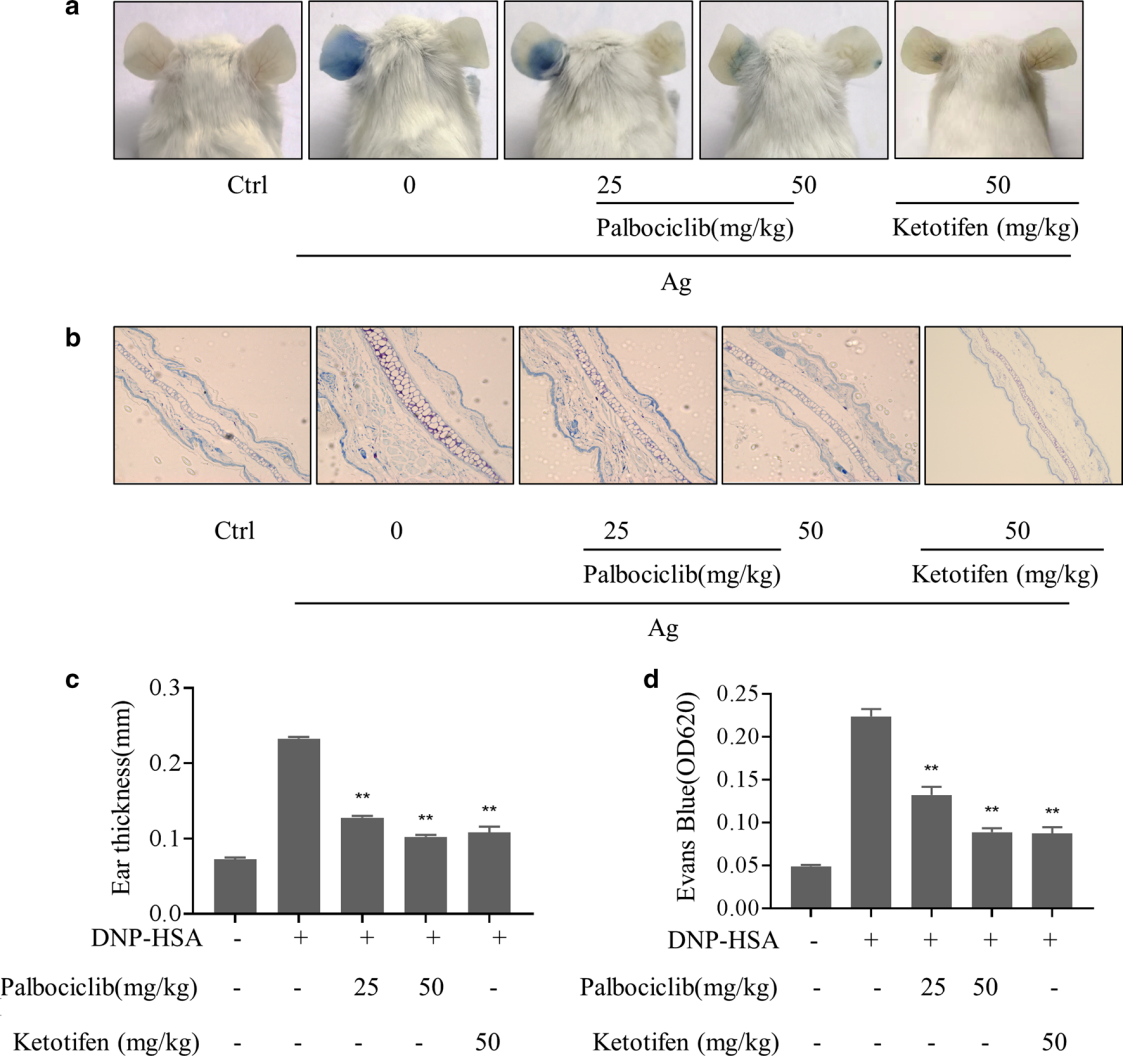
Palbociclib suppression of PCA in vivo. Mouse ear skin (N = 5/group) was sensitized with anti-DNP IgE, treated (or not) with palbociclib, and injected with 20 μg DNP-HSA containing 1% Evans blue as described in detail in “Materials and methods”. Ketotifen treatment was as positive control. **a** Representative images of PCA mouse ears. **b** Representative photomicrographs of PCA ear tissue sections. Palbociclib reverses PCA-induced ear thickening (**c**) and PCA-induced increases in Evans Blue OD_620nm_. Means ± SDs of 3 independent experiments are shown; *p < 0.05, **p < 0.01 vs. control

Inhibition of mast cell activation was confirmed in a positive control group treated with the mast cell stabilizer ketotifen. When ears were injected with 4% Evans blue dye mixed with antigen in PCA tests, they showed marked thickening in response and Evans blue solution poured out from PCA reaction sites, demonstrating vascular hyperpermeability. Dye solution extravasation and ear thickening were inhibited by 25 mg/kg or 50 mg/kg palbociclib (Fig. 4).

### Palbociclib attenuated ASA in vivo

**Fig. 5:**
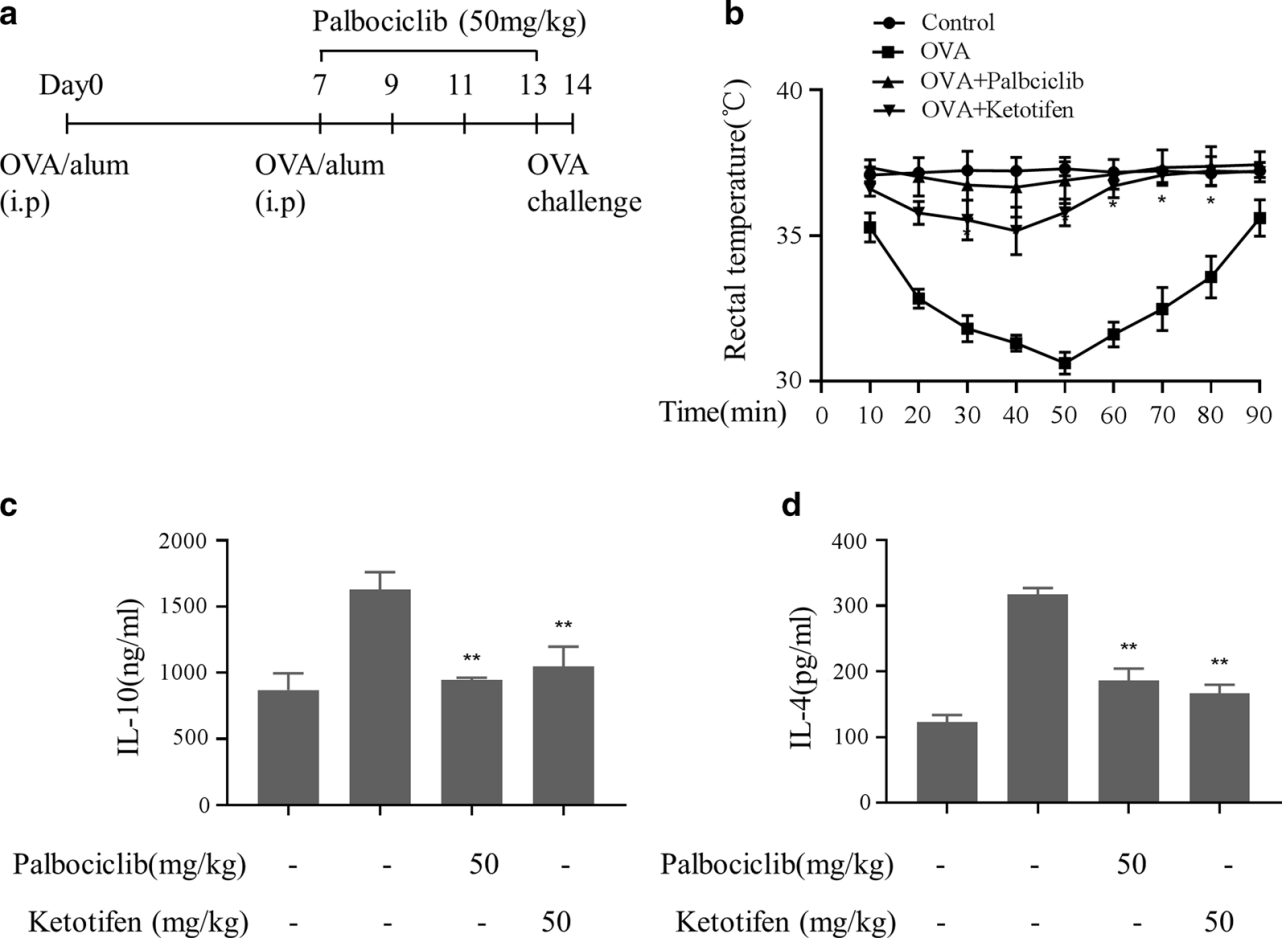
Palbociclib suppression of ASA in vivo. **a** ASA model protocol (N = 5/group). Ketotifen treatment was as positive control. Palbociclib prevents ASA-induced reductions in body temperature **b** as well as ASA-induced increases in IL-10 (**c**) and IL-4 (**d**) serum levels in ASA mice (determined by ELISA). Means ± SDs of 3 independent experiments are shown; *p < 0.05, **p < 0.01 vs. control

Mice were sensitized by repeated administration of OVA with alum adjuvant, and anaphylaxis was induced with an intraperitoneal OVA challenge, as shown in Fig. 5a. Ketotifen treatment was as positive control. OVA mice exhibited decreasing rectal temperatures 30–50 min after the OVA challenge injection, and these temperature reductions were attenuated by palbociclib (Fig. 5b). Concomitantly, total serum IL-4 and IL-10 levels reflective of inflammation were increased after the OVA challenge and those increases were suppressed by palbociclib (Fig. 5c, d).

## Discussion

In the present study, palbociclib exhibited inhibitory effects on mast cell degranulation, as evidenced by reduced release of histamine and β-hexosaminidase by DNP-IgE/HAS-stimulated BLCs and BMMCs. In our in vivo experiments, palbociclib attenuated the DNP-IgE/HSA-induced PCA reaction (Evans blue extravasation) dose-dependently and suppressed ASA responses (i.e. OVA challenge-induced body temperature reduction and serum IL-4 or IL-10 level increases). Together, these results show that the CDK4/6 inhibitor palbociclib can suppress IgE-mediated mast cell activation in vitro and in vivo.

During mast cell degranulation, morphological changes occur due to the actions of contractile microfilaments [29]. Mast cell activation via aggregation of IgE-FcεRI complexes causes degranulation and release of proinflammatory mediators, and these processes involve F-actin reorganization [25, 29]. Our findings showing that pretreatment with palbociclib inhibited cell shape changes and cytoskeletal decomposition in BLCs resemble previously reported inhibitory effects of coptisine on mast cell degranulation [24].

Activation of protein tyrosine kinases is the earliest detectable signaling response to FcεRI cross-linking on mast cells [26]. Upon tyrosine kinase activation, molecular mechanisms involving the Src family kinase Lyn [30] and/or intracellular signaling via MAPKs [26, 31] promotes mast cell degranulation. Mast cell activation inhibitors, including the corticosteroid dexamethasone [21], have been shown to suppress mast cell activation by down-regulating signaling pathways. Here, similarly, we observed that palbociclib suppressed Lyn activation and MAPK pathway signaling.

Drug repurposing, wherein previously developed pharmacotherapies are used in new applications, can accelerate clinical drug discovery and development [32]. The major advantages of drug repurposing are previously established low cytotoxicity and pharmacokinetic activity [33]. For example, metformin, a widely used anti-diabetic drug [34] that also has anti-cancer effects [35–37], has been shown to inhibit IgE and aryl hydrocarbon receptor-mediated mast cell activation in vitro and in vivo [38]. Additionally, the antidiarrheal medicine berberine, which is widely used for gastrointestinal ailments such as bacterial gastroenteritis and dysentery [39], has been found to suppress mast cell-mediated allergic responses via down-regulation of FcɛRI activation and MAPK signaling [40]. Hence, searching among existing drugs for pharmacotherapies that can be repurposed into novel allergic disease treatments that target mast cell activation represents a promising strategy.

Currently, palbociclib is being used as an anti-cancer drug. It inhibits the growth of cancer cells by inhibiting CDK4/6. In 2017, it was approved by the US FDA as an adjuvant therapy, with endocrine blockers, for ER+/HER2− advanced breast cancer in postmenopausal women. Pooled safety analysis (PALOMA trial) revealed a peak incidence of adverse events in the first 6 months of treatment, with a subsequent decrease in incidence over time [41]. In clinical use, oral palbociclib is generally well-tolerated at its oncological dosage of 125 mg daily on a 21-day on, 7-day off schedule [42, 43]. When administered at a dose of 150 mg/kg, palbociclib does not affect body weight in mice [44]. Palbociclib’s low toxicity profile and ability to inhibit mast cell activation in our murine models suggest that palbociclib could be used to alleviate IgE-mediated allergic diseases in human patients.

Palbociclib inhibition of cell growth by way of cell cycle arrest [45, 46], suggests that mast cell activation may require unimpeded cell cycle progression. Upregulation of CDK6 expression can enhance MAPK and NF-κB signaling [47, 48], both of which have been associated with mast cell activation [26]. Previous demonstrations showing that palbociclib can inhibit neoplastic mast cell proliferation [49] did not indicate whether palbociclib also inhibits mast cell activation, and specifically cell degradation, a key allergic disease treatment target. Hence, given palbociclib’s CDK inhibitory actions and its ability to suppress signaling molecule expression and/or activation, we hypothesize that palbociclib administered at a low-cytotoxicity dose may exert anti-IgE-mediated allergy effects by down-regulating of MAPK and/or NF-κB signaling downstream of CDK6 inhibition via FcεRI, whose activation may be modulated by the Src family kinase Lyn [30]. Lyn, interacting with FcεRIβ, is indispensable for FcεRI-mediated human mast cell activation, and specific inhibition of Lyn signaling may represent a new therapeutic strategy for the treatment of human allergic diseases [50]. Our results showed that palbociclib inhibition of mast cell activation may involve suppression of Lyn and/or MAPK signaling.

Interestingly, palbociclib reduced the total serum IL-10 levels in the ASA model. IL-10 is secreted by a wide variety of cell types, even including mast cells [51]. IL-10 enhances IgE-mediated mast cell responses and is necessary mediator of allergy development in vivo [51]. Additionally, IL-10 has been shown to be critical for Th2 responses in a murine allergy model [52]. The present results suggest that palbociclib inhibition of mast cells may reduce IL-10 levels, or even Th2 cells. These possibilities need to be examined directly with further experimentation.

In conclusion, our findings showing that the CDK4/6 inhibitor palbociclib can suppress IgE-mediated mast cell activation in vitro and in vivo suggest that palbociclib is a potential therapeutic candidate for treating mast cell-mediated allergic diseases, including allergic rhinitis, atopic dermatitis, and anaphylaxis [27, 28]. Inhibition of mast cell activation—an important pathogenic process in IgE-induced allergic reactions [11]—represents a novel strategy for relieving allergic symptoms and treating allergic reactions.

## Data Availability

The datasets used and/or analyzed during the current study are available from the corresponding author on reasonable request.
